# Factors associated with perceived resilience among undergraduate nursing students: findings of the first cross-sectional study in Palestine

**DOI:** 10.1186/s12912-023-01325-6

**Published:** 2023-05-05

**Authors:** Aidah Alkaissi, Nizar B. Said, Shorooq Qadous, Mahdia Alkony, Omar Almahmoud

**Affiliations:** 1 Nursing and Midwifery Department, Faculty of Medicine and Health Sciences, An-Najah National University, Nablus, Palestine; 2 Nursing and Health Professions College, Birzeit University, Birzeit, Palestine

**Keywords:** Nursing education, Nursing students, Resilience, Psychological well-being

## Abstract

**Background:**

Resilience has emerged as a concept that could explain and predict good academic and well-being of students in stressful and traumatic situations. This study was conducted to assess resilience and identify predictors of high or low resilience scores among future nurses in Palestine.

**Methods:**

This cross-sectional study adhered to the Strengthening the Reporting of Observational Studies in Epidemiology statement. Undergraduate nursing students in all academic years were recruited and asked to complete a questionnaire that contained the Trait Resilience Scale and the State-Resilience Scale. Multiple linear regression models were used to identify predictors of higher resilience scores and to control for potentially confounding factors.

**Results:**

In this study, 290 students completed the questionnaire (response rate = 92.4%). The means of trait, state, and combined resilience scores were 71.4 (SD: 7.5), 62.7 (SD: 6.7), and 134.1 (SD: 12.8), respectively. There was a significant moderate positive correlation between state resilience scores and trait resilience scores (*r* = 0.63, *p* < 0.001). Having a study routine on daily basis predicted higher trait (β = -0.20, *p *< 0.001), state (β = -0.12, *p* = 0.032), and combined (β = -0.18, *p* = 0.001) resilience scores. Trait and combined resilience scores were predicted by the absence of chronic diseases (β = 0.12, *p* < 0.05), experiencing addiction issues (β = -0.11, *p* < 0.05), living in Israeli seized areas (β = 0.16, *p* < 0.05), and living in a house with enough number of rooms per siblings (β = 0.13, *p < *0.05). On the other hand, state and combined resilience scores were predicted by being in the first academic year (β = -0.18, *p* < 0.01), and state resilience scores were predicted by living in urban areas (β = -0.12, *p* < 0.05).

**Conclusions:**

Undergraduate nursing students in Palestine reported relatively high trait and state resilience scores. Higher trait, state, and combined resilience scores were predicted by having a study routine on daily basis. More studies are still needed to investigate the relationship between resilience scores, perceived well-being, willingness to care, and the future success of nursing students in Palestine.

## Background

Nurses are one of the main healthcare providers in all healthcare systems around the world. In different healthcare systems, nurses provide the largest volume of healthcare services to patients who visit primary, secondary, and tertiary healthcare centers [1]. Currently, there is a shortage of nurses in many healthcare systems around the globe [2]. Therefore, nursing schools are under continuous pressure to provide quality education and smooth transition of nursing students to their future roles in professional practice. Nurses as well as future nurses are hard-pressed to maintain professional standards with increasing specializations in healthcare delivery, more involvement of patients in their healthcare, and tensions between demand and available resources [2–4]. Globally, there is a large number of nursing students who experience struggles to complete their nursing education [5].

Several studies have shown that nursing students face higher levels of academic stress compared to students in other fields of healthcare [6, 7]. During their on-site training, nursing students have to attend long training hours, witness the sufferings and death of patients, and risk exposure to contagious diseases [8, 9]. Additionally, nursing students have to reconsider their personal views and values as they use new concepts and skills they acquire during training. Previous studies have shown that these situations could be highly stressful [10]. Because of their underdeveloped coping abilities and lack of experience in dealing with conflict situations, nursing students could be at higher risk for detrimental consequences of stress [11].

Resilience has emerged as a term that is commonly utilized to describe the ability of individuals to turn adversity into opportunities and learn from different demanding situations [12]. Resilience was defined as the ability of someone to overcome adversity, retain a sense of control over their environments, maintain equilibrium, and continue to move on in a positive manner [13, 14]. As a trait, resilience can be conceptualized as a heritable characteristic, distinctive quality, strength, and/or aspect of someone’s personality that could be relatively stable over time [13]. On the other hand, resilience can also be conceptualized as a state which refers to affective, motivational, and cognitive potentials that could be relatively malleable and adaptive in different social-ecological contexts. Resilience has emerged as a concept that could explain and predict good academic and well-being of students in stressful and traumatic situations [13, 14]. Individuals who developed resilience typically report high self-esteem, perceived well-being, and greater flexibility in dealing with difficult situations compared to individuals with low resilience [15]. Resilience is an essential trait that practicing nurses and future nurses should possess. Previous studies investigated resilience among nursing students [5, 11, 16, 17]. In a recent systematic review of 12 studies, Li and Hasson reported moderate resilience and high-stress levels that caused negative psychological health outcomes among nursing students [11]. High resilience and low stress predicted the well-being of nursing students. In Hong Kong, Chow et al. showed that higher resilience scores predicted the perceived well-being of nursing students [16]. In another study, Van Hoek et al. showed that higher resilience predicted academic success and low dropout rates among nursing students [18]. Additionally, resilience was shown to contribute to nursing students’ readiness to provide care to patients [5]. It has been argued that future healthcare professionals need to be prepared to deal with the emotional and physical stress experienced during their training [17]. Consistently, all studies reviewed by Li and Hasson recommended informing decisions and policymakers in academia to improve resilience, well-being, and reduce stress among nursing students [11]. It has been argued that promoting resilience among nursing students can help them achieve academic success and ensures their smooth transition into their future professional practice [19]. In a systematic review with thematic synthesis, Amsrud et al. qualitatively synthesized evidence on how educators can support nursing students to develop resilience [5]. The study concluded that a learning culture that is characterized by trustworthiness might promote resilience among nursing students.

Little is known about the level of resilience among undergraduate nursing students in Palestine. Assessing resilience among nursing students can inform decisions and policymakers in academia to improve learning culture, well-being, academic success, and smooth transition of nursing students into their future professional practice roles. Therefore, this study aimed to assess resilience among undergraduate nursing students in Palestine and to identify predictors of high or low resilience among future nurses.

## Methods

### Study design and setting

This study was conducted in a cross-sectional design among undergraduate nursing students in the largest and main university with a nursing program in Palestine. The study adheres to the Strengthening the Reporting of Observational Studies in Epidemiology (STROBE) statement.

The undergraduate nursing program in Palestine consists of 136 credit hours that can be completed in 4 academic years. Graduates are the future workforce of nurses who assume professional roles in primary, secondary, and tertiary healthcare practice.

### Study population, sample size, inclusion, and exclusion criteria

The study population was undergraduate nursing students in the largest teaching university in Palestine. At the time of the study, there were approximately 1,200 students in the undergraduate nursing program. The sample size needed for this study was estimated using Daniel’s formula [20]. An online sample size calculator (http://www.raosoft.com/samplesize.html) was used to calculate the sample size at a 95% confidence interval (CI) with a maximal margin of error of 5%. The sample size needed for this study was 292 undergraduate nursing students. To account for potential dropouts, we invited more than the sample size needed for this study.

The study participants were included in this study when they met the following inclusion criteria: 1) a current student in an undergraduate nursing program, 2) willing to provide informed consent, and 3) willing to respond to items in a questionnaire. Students who were in postgraduate nursing programs and those who already completed their undergraduate nursing program were excluded from this study.

### The study tool

**Fig. 1:**
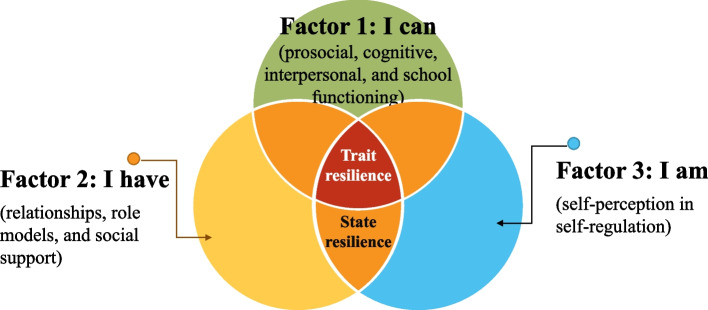
Theoretical framework

The bulk of the accumulating literature refers to resilience as a psychological adjustment or an adaptive capacity [13, 21]. Resilience is thought to be promoted by cultural, social, and physical factors. Additionally, resilience is also affected by the extent to which a person negotiates for using these factors. Therefore, resilience is thought to be more than an outcome of person, process, risk, and context interaction [13]. Previous studies have reported that resilience was associated with self-efficacy, coping, mindfulness, and psychological adjustment [13, 21]. Self-efficacy refers to one’s belief that one can perform a specific task. It has been argued that behaviors are continuous interactions between environmental, behavioral, and cognitive factors. These interactions might vary by experience. Coping is the process in which one adjusts after an adverse event. People cope either by making efforts to solve the problem, reduce the emotional tensions that are associated with the adverse event, or tend to refrain from facing a similar adverse event. Mindfulness is the ability to attain a de-centered perspective on an event and respond flexibly to a negative thought. In this study, the Trait Resilience Scale was selected because it was based on a three-dimensional model of trait resilience (Factor 1: I can, Factor 2: I have, and Factor 3: I am) [22, 23]. Factor 1 measured the prosocial, cognitive, interpersonal, and school functioning of the students. Factor 2 measured relationships, role models, and social support the student had. Factor 3 measured self-perception in the self-regulation of the students. Additionally, the State-Resilience Scale was selected because it was based on a two-dimensional model of state resilience (Factor 1: I am and Factor 2: I have) [22, 23]. A theoretical framework is shown in Fig. 1. The scales were originally developed to measure resilience among students. In this study, the scales were used with permission from the developers to measure trait and state resilience among undergraduate nursing students.

The questionnaire that was used in this study consisted of 3 sections. The first section collected the sociodemographic, economic, and academic variables of the nursing students like gender, age, smoking status, place where the student was born and raised, where the student lived, whether the student needed to commute to the university or not, whether the student lived in a dormitory/residence or with parents, whether parents were divorced/separated, parents’ level of education, whether the student lived in a family-owned house or rented house, if the house had enough room per number of siblings or not, household income, whether the student worked a part-time job or not, whether the student had a chronic disease or not, whether the student experienced addiction issues or not, academic year, grade point average (GPA), study routine, and the number of hours of social media connection per day. In Palestinian universities, a GPA of 2 in the letters system is equivalent to C. Grades are converted to letters as follows: A: ≥ 90%, 88.0% ≤ A- < 90.0%, 85.0% ≤ B +  < 88.0%, 80.0% ≤ B < 85.0%, 78.0% ≤ B- < 80.0%, 74.0% ≤ C +  < 78.0%, 70.0% ≤ C < 74.0%, 65.0% ≤ C- < 70.0%, 63.0% ≤ D +  < 65.0%, 60.0% ≤ D < 63.0%, 45.0% ≤ D- < 60.0%, and E < 45.0%. These variables were shown to affect resilience among nursing and other healthcare students [13, 21, 24–26]. The second section contained the Trait Resilience Scale which consisted of 18 items [22, 23]. The students had to rate each item of the scale on a Likert scale that had 5 options (strongly disagree to strongly agree). The third section contained the State-Resilience Scale which consisted of 15 items [22, 23]. The students had to rate each item of the scale on a Likert scale that had 5 options (strongly disagree to strongly agree). The ratings that were collected on the 5-options Likert scale were transformed into scores as: strongly disagree = 1, disagree = 2, neutral = 3, agree = 4, and strongly agree = 5. Scores on the Trait Resilience Scale could range from 18–90. Students were stratified into trait resilience categories based on their scores as: very low (score: 15–24), low (score: 25–34), moderate (score: 35–44), high (score: 45–54), very high (score: 55–64), and extreme (score: 65–75) [22, 23]. Scores on the State-Resilience Scale could range from 15–75. Students were also stratified into state resilience categories based on their scores as: very low (score: 18–29), low (score: 30–41), moderate (score: 42–53), high (score: 54–65), very high (score: 66–77), and extreme (score: 78–90) [22, 23]. Historically, resilience has emerged as a trait and/or a state. Because of this dualistic approach to analyzing resilience, different scales were developed that operationalized resilience as either a trait or a state. In their recent work, Kuldas and Foody (2022) have called for a transactional approach to define resilience instead of the commonly used dualistic approach [13]. Because higher scores indicated higher resilience in both scales, a combined “composite” score was computed by adding both Trait Resilience Scale and State-Resilience Scale scores.

The questionnaire was assessed for face validity by 4 academicians who were also registered nurses. Before the questionnaire was used in the larger study, the questionnaire was pilot tested among 15 undergraduate nursing students. The test–retest method was used to assess the stability of scores over a short period. The nursing students were asked to complete the questionnaire. After 30 min to 2 h, the same nursing students were asked to complete the questionnaire once again. Scores obtained in both rounds were correlated using Pearson’s correlations. The stability of scores was ensured by a Pearson’s coefficient (r) of > 0.80. In this study, the value of r was 0.90 which indicated excellent stability of scores. The internal consistencies of the items in the State-Resilience Scale and Trait Resilience Scale were assessed using Cronbach’s alpha statistics. A Cronbach’s alpha value of > 0.70 was considered acceptable. In this study, the 18 items in the Trait Resilience Scale had a Cronbach’s alpha of 0.78 and the 15 items in the State-Resilience Scale had a Cronbach’s alpha of 0.77. These values indicated that the items in both scales were internally consistent.

The tool used in this study was a paper-based questionnaire that was distributed to the nursing students by the class representative of each session (academic year). The class representatives were also students who were elected by the other students to represent them in organizing academic and social activities. The participants were informed that participation was voluntary and anonymous as no data relevant to their identity would be collected. The participants were also informed that answering every item was necessary, otherwise, the participants should not return the questionnaire. English is the language of instruction in the nursing programs taught in Palestine. Courses are taught and examinations are held in the English language. Therefore, the questionnaire was originally developed in English. To avoid any confusion, each item was translated into Arabic language. The items were translated by the researchers who were fluent in both Arabic and English. To ensure the accuracy of the translation, forward- and back-translations were used.

### Data collection and analysis

The researchers approached the class representatives and explained the objectives of the study to them. The class representatives were provided with additional written information about the objectives and design of the study. The class representatives were asked to invite the other students to participate in this study. Students who expressed willingness to provide informed consent were asked to complete the questionnaire.

The data collected in this study were entered into Statistical Package for the Social Sciences version 21.0 for Windows. Absolute skewness and kurtosis were used to assess the data for the normality of distribution. As the absolute skewness was within the range of -2.0 and + 2.0 and the absolute kurtosis was in the range of -7.0 and + 7.0, the data were considered normally distributed [27, 28]. In this study, the data were expressed as means with their corresponding standard deviation (SD) values. Differences in resilience scores among the nursing students were compared using Student’s *t*-test. Scores were correlated using Pearson’s correlations. High correlations were indicated by Pearson’s correlation coefficients (r) of ≥ 0.70 and moderate correlations were indicated by 0.50 ≤ r < 0.70 [29]. Multiple linear regression models were used to identify predictors of higher resilience scores and to control for potentially confounding factors. In this study, the variables with a p of < 0.10 in the Student’s *t*-test were retained in the regression model. Goodness-of-fit was evaluated using the adjusted R-squared (R^2^) with a p of < 0.05. Durbin-Watson statistics were used to detect autocorrelation problems. Durbin-Watson in the range of 1.5 to 2.5 indicated the absence of autocorrelation issues. The variance inflation factor (VIF) and tolerance values were used to ensure the absence of multicollinearity problems. In this study, tolerance values ≥ 0.25 and VIF values ≤ 2.5 were considered acceptable and indicated the absence of multicollinearity problems [30]. A p of < 0.05 indicated statistical significance.

### Ethical approval

An-Najah National University's Institutional Review Board (IRB) approved this study. The study was compliant with the Declaration of Helsinki on the ethical principles for human medical research. Participation in this study was completely voluntary. All participants provided informed consent. Data were collected anonymously and no information leading to the identity of the participant was collected in this study.

## Results

### Study participants

In this study, 314 undergraduate nursing students were invited by the class representatives. Of those, 290 students completed the questionnaire, giving a response rate of 92.4%. In this study, 129, 72, 71, and 42 students were invited from the first, second, third, and fourth academic year. The response rates were 96.1%, 93.1%, 88.7%, and 85.7%, respectively. About half of the nursing students were male and 62.4% were 20 years and older. The majority (79.7%) of the nursing students were nonsmokers, 73.8% were born and raised in the occupied Palestinian territories, and 74.8% lived in rural areas. Of the nursing students, 53.1% lived with their parents and needed to commute to the university. The vast majority (96.2%) of the students lived with their parents who had their own family house (94.1%). Of the nursing students, 41.4% stated that the house had enough rooms per number of siblings. Of the students, 28.3% stated that their parents attended a university and were able to make some savings. About 1 in every 5 nursing students worked a part-time job, 5.2% had a chronic disease, and 3.4% experienced addiction issues. Of the students, 42.8% were in their first academic year, 61.4% had a GPA of 2 or more, 37.2% had a study routine every day, and 7.9% stayed connected to social media for 9 or more hours per day. Details of the sociodemographic, economic, and academic variables of the nursing students are provided in Table 1.

**Table 1:** Sociodemographic, economic, and academic variables of the nursing students (*n* = *290*)

**Variable**	**n**	**%**
**Gender**		
Male	146	50.3
Female	144	49.7
**Age (years)**		
≥ 20	109	37.6
< 20	181	62.4
**Smoking status**		
Smoker	59	20.3
Nonsmoker	231	79.7
**Place where the student was born and raised**		
Occupied Palestinian territories	214	73.8
Palestinians living in Israeli areas	76	26.2
**Living area**		
Urban	73	25.2
Rural	217	74.8
**Need to commute to the university**		
Yes	154	53.1
No	136	46.9
**Living in a dormitory/residence**		
Yes	136	46.9
No	154	53.1
**Divorced/separated parents**		
Yes	11	3.8
No	279	96.2
**Parents' level of education**		
School	208	71.7
University	82	28.3
**Lived in a family-owned house/rented house**		
Family-owned house	273	94.1
Rented house	17	5.9
**The house had enough rooms per number of siblings**		
No	170	58.6
Yes	120	41.4
**Household income**		
Enough to cover everyday life expenses	208	71.7
Able to make some savings	82	28.3
**Worked a part-time job**		
Yes	55	19.0
No	235	81.0
**Presence of a chronic disease**		
Yes	15	5.2
No	275	94.8
**Experience addiction issue**		
Yes	10	3.4
No	280	96.6
**Academic year**		
First	124	42.8
Second	67	23.1
Third	63	21.7
Fourth	36	12.4
**Grade point average (GPA)**		
≥ 2	112	38.6
< 2	178	61.4
**Study routine**		
Every day	108	37.2
Not every day	180	62.1
**Number of hours of social media connection**		
≥ 9	267	92.1
< 9	23	7.9

### Resilience scores

The mean trait resilience score was 71.4 (SD: 7.5), the mean state resilience score was 62.7 (SD: 6.7), and the mean combined resilience score was 134.1 (SD: 12.8). There was a significant moderate positive correlation between state resilience scores and trait resilience scores (*r* = 0.63, *p* < 0.001). The distribution of ratings of the nursing students on the 18-item trait scale and 15-item state resilience scale are shown in Table 2.

**Table 2:** Distribution of ratings of the nursing students on the 18-item trait scale and 15-item state resilience scale

			**Strongly disagree**		**Disagree**		**Neutral**		**Agree**		**Strongly agree**	
**Resilience**	**#**	**Item**	**n**	**%**	**n**	**%**	**n**	**%**	**n**	**%**	**n**	**%**
**Trait**	1	I am expected to be a helpful person	0	0.0	0	0.0	24	8.3	114	39.3	152	52.4
	2	I am calm even in times of difficulties	27	9.3	49	16.9	92	31.7	82	28.3	40	13.8
	3	Others see me as alert and physically active	5	1.7	12	4.1	72	24.8	138	47.6	63	21.7
	4	I believe in myself	4	1.4	7	2.4	24	8.3	87	30.0	168	57.9
	5	My parents give me attention	5	1.7	11	3.8	51	17.6	85	29.3	138	47.6
	6	My family has high expectations of me	3	1.0	5	1.7	28	9.7	95	32.8	159	54.8
	7	When I am upset or in trouble, there is usually someone that I can turn to	25	8.6	31	10.7	76	26.2	94	32.4	64	22.1
	8	I am successful in school	1	0.3	4	1.4	26	9.0	112	38.6	147	50.7
	9	I actively do things to help others	2	0.7	10	3.4	64	22.1	115	39.7	99	34.1
	10	I feel that I understand myself	12	4.1	15	5.2	57	19.7	109	37.6	97	33.4
	11	I am exposed to stressful difficulties that I learned to handle	7	2.4	27	9.3	100	34.5	109	37.6	47	16.2
	12	I feel that things will turn out well even in difficult situations	5	1.7	12	4.1	76	26.2	131	45.2	66	22.8
	13	I know how to plan for the future	3	1.0	23	7.9	69	23.8	125	43.1	70	24.1
	14	Others usually seem happy to see me	0	0.0	3	1.0	58	20.0	126	43.4	103	35.5
	15	My parents tell me that I am good-natured and easy-going	11	3.8	15	5.2	57	19.7	129	44.5	78	26.9
	16	I have warm positive relationships with adults	3	1.0	10	3.4	39	13.4	131	45.2	107	36.9
	17	I am persistent in my actions till I succeed	1	0.3	7	2.4	70	24.1	115	39.7	97	33.4
	18	I am able to figure out effective ways of dealing with problems	2	0.7	21	7.2	80	27.6	123	42.4	64	22.1
**State**	1	I have someone who loves me	10	3.4	5	1.7	20	6.9	57	19.7	198	68.3
	2	I have a person outside my home who I can tell about my problems or feelings	19	6.6	19	6.6	51	17.6	88	30.3	113	39.0
	3	I am praised for doing things on my own	8	2.8	7	2.4	43	14.8	122	42.1	110	37.9
	4	I can count on my family being there when needed	4	1.4	14	4.8	45	15.5	113	39.0	114	39.3
	5	I have someone who I want to be like (a role model)	11	3.8	14	4.8	54	18.6	82	28.3	129	44.5
	6	I believe things will turn out alright	4	1.4	9	3.1	51	17.6	118	40.7	108	37.2
	7	I do endear things that make people like me	1	0.3	13	4.5	63	21.7	119	41.0	94	32.4
	8	I have faith in a higher being	0	0.0	3	1.0	25	8.6	51	17.6	211	72.8
	9	I am willing to try new things	2	0.7	2	0.7	27	9.3	106	36.6	153	52.8
	10	I like to achieve in what I do	2	0.7	3	1.0	23	7.9	90	31.0	172	59.3
	11	I feel that what I do makes a difference in how things turn out	0	0.0	9	3.1	50	17.2	128	44.1	103	35.5
	12	I like myself	7	2.4	7	2.4	37	12.8	111	38.3	128	44.1
	13	I can focus on a task and stay with it	2	0.7	13	4.5	60	20.7	121	41.7	94	32.4
	14	I have a sense of humor	3	1.0	13	4.5	52	17.9	115	39.7	107	36.9
	15	I make plans to do things	4	1.4	21	7.2	41	14.1	108	37.2	116	40.0

The majority of the nursing students reported very high or extreme trait resilience (79.0%) and state resilience (87.6%). The distribution of the students in trait and state resilience categories is shown in Table 3.

**Table 3:** Distribution of the students in trait and state resilience categories

**Resilience**	**Category**	**n**	**%**
**Trait**	Very low (score: 15–24)	0	0.0
	Low (score: 25–34)	0	0.0
	Moderate (score: 35–44)	5	1.7
	High (score: 45–54)	56	19.3
	Very high (score: 55–64)	165	56.9
	Extreme (score: 65–75)	64	22.1
**State**	Very low (score: 18–29)	0	0.0
	Low (score: 30–41)	0	0.0
	Moderate (score: 42–53)	3	1.0
	High (score: 54–65)	33	11.4
	Very high (score: 66–77)	131	45.2
	Extreme (score: 78–90)	123	42.4

### Association between sociodemographic, economic, and academic variables of the students with resilience scores

The mean trait, state, and combined resilience scores were significantly higher for undergraduate nursing students who lived in Israeli seized areas compared to those who lived in occupied Palestinian territories, reported higher household income compared to those who reported higher household income compared to those who reported lower household income, were in the first academic year compared to those who were in the second-fourth academic year, and reported a study routine on daily basis compared to those who did not report a study routine daily as shown in Table 4. The mean trait and combined resilience scores were significantly higher for undergraduate nursing students who lived in a house with enough rooms per number of siblings compared to those who lived in a house without enough rooms per number of siblings. The mean state and combined resilience scores were significantly higher for undergraduate nursing students who lived in urban areas compared to those who lived in rural areas, did not need to commute to the university compared to those who needed to commute to the university, and those who lived in a dormitory/residence compared to those who did not live in a dormitory/residence. The mean trait scores were significantly higher for undergraduate nursing students who had a university education compared to those who had a school education and those who did not have chronic diseases compared to those who had chronic diseases. Differences in the mean resilience scores are shown in Table 4.

**Table 4:** Association between sociodemographic, economic, and academic variables of the students with state and trait resilience scores

			**Trait resilience score**			**State resilience score**			**Combined resilience score**		
**Variable**	**n**	**%**	**Mean**	**SD**	**p**	**Mean**	**SD**	**p**	**Mean**	**SD**	**p**
**Gender**											
Male	146	50.3	71.5	7.7	0.737	61.9	7.0	0.042	133.4	13.1	0.392
Female	144	49.7	71.2	7.4		63.5	6.2		134.7	12.5	
**Age (years)**											
≥ 20	109	37.6	71.1	7.0	0.554	63.4	6.8	0.159	134.4	12.6	0.701
< 20	181	62.4	71.6	7.9		62.2	6.6		133.8	13.0	
**Smoking status**											
Smoker	59	20.3	72.3	8.1	0.307	63.1	7.4	0.619	135.3	14.1	0.390
Nonsmoker	231	79.7	71.2	7.4		62.6	6.5		133.7	12.5	
**Place where the student was born and raised**											
Occupied Palestinian territories	214	73.8	70.2	7.6	< 0.001	61.8	6.5	< 0.001	132.0	12.6	< 0.001
Palestinians living in Israeli areas	76	26.2	74.7	6.3		65.1	6.6		139.9	11.5	
**Living area**											
Urban	73	25.2	72.2	7.7	0.280	64.7	6.1	0.003	136.9	12.2	0.028
Rural	217	74.8	71.1	7.5		62.0	6.7		133.1	12.9	
**Need to commute to the university**											
Yes	154	53.1	70.9	7.6	0.265	61.7	6.5	0.009	132.6	12.7	0.044
No	136	46.9	71.9	7.5		63.8	6.7		135.7	12.8	
**Living in a dormitory/residence**											
Yes	136	46.9	72.1	7.2	0.113	63.7	6.7	0.013	135.8	12.5	0.026
No	154	53.1	70.7	7.8		61.8	6.5		132.5	12.9	
**Divorced/separated parents**											
Yes	11	3.8	68.5	7.7	0.188	61.5	8.6	0.571	130.0	16.0	0.285
No	279	96.2	71.5	7.5		62.7	6.6		134.2	12.7	
**Parents' level of education**											
School	208	71.7	70.8	7.3	0.028	62.6	6.8	0.900	133.4	12.8	0.175
University	82	28.3	72.9	7.9		62.7	6.4		135.7	12.8	
**Lived in a family-owned house/rented house**											
Family-owned house	273	94.1	71.4	7.5	0.851	62.6	6.6	0.802	134.1	12.7	0.984
Rented house	17	5.9	71.1	8.4		63.1	7.9		134.1	15.1	
**The house had enough rooms per number of siblings**											
No	170	58.6	70.3	7.6	0.003	62.1	6.6	0.067	132.3	12.9	0.007
Yes	120	41.4	73.0	7.1		63.5	6.6		136.5	12.4	
**Household income**											
Enough to cover everyday living expenses	208	71.7	70.8	7.6	0.020	62.1	6.7	0.018	132.8	13.1	0.009
Able to make some savings	82	28.3	73.0	7.2		64.1	6.2		137.2	11.5	
**Worked a part-time job**											
Yes	55	19.0	72.6	7.6	0.174	63.3	6.6	0.401	136.0	12.9	0.216
No	235	81.0	71.1	7.5		62.5	6.7		133.6	12.8	
**Presence of a chronic disease**											
Yes	15	5.2	67.6	11.1	0.045	60.7	8.0	0.233	128.3	17.2	0.072
No	275	94.8	71.6	7.3		62.8	6.6		134.4	12.5	
**Experience addiction issue**											
Yes	10	3.4	75.9	7.2	0.054	64.9	7.6	0.281	140.8	12.3	0.090
No	280	96.6	71.2	7.5		62.6	6.6		133.8	12.8	
**Academic year**											
First-year	124	42.8	72.4	7.1	0.047	65.1	6.3	< 0.001	137.5	12.2	< 0.001
Second year and beyond	166	57.2	70.6	7.8		60.9	6.4		131.5	12.7	
**Grade point average (GPA)**											
≥ 2	112	38.6	70.8	7.2	0.291	63.0	7.3	0.454	133.8	13.6	0.817
< 2	178	61.4	71.8	7.7		62.4	6.2		134.2	12.3	
**Study routine**											
Every day	108	37.2	73.2	7.5	0.001	64.0	6.5	0.010	137.2	12.8	0.010
Not every day	182	62.8	70.3	7.4		61.9	6.7		132.2	12.5	
**Number of hours of social media connection per day**											
≥ 9	267	92.1	71.6	7.5	0.166	62.6	6.7	0.633	134.2	12.9	0.572
< 9	23	7.9	69.3	8.2		63.3	6.6		132.6	12.3	

The multiple linear regression models showed that higher trait, state, and combined resilience scores were predicted by having a study routine on daily basis. Trait and combined resilience scores were predicted by the absence of chronic diseases, experiencing addiction issues, living in Israeli seized areas, and living in a house with enough rooms per sibling. On the other hand, state and combined resilience scores were predicted by being in the first academic year and state resilience scores were predicted by living in urban areas. Details of the multiple linear regression analyses are shown in Table 5.

**Table 5:** Multiple linear regression of associations between sociodemographic, economic, and academic variables of the students with state and trait resilience scores

**Resilience**	**Variable**	**B**	**SE**	**β**	**t**	**p**	**Tolerance**	**VIF**
**Trait**^**a**^	Academic year	-0.85	0.90	-0.06	-0.95	0.345	0.88	1.14
	Presence of a chronic disease	3.91	1.89	0.12	2.06	0.040	0.97	1.03
	Experience addiction issue	-4.65	2.35	-0.11	-1.98	0.049	0.92	1.09
	Divorced/separated parents	0.89	2.21	0.02	0.40	0.688	0.96	1.04
	Living in a dormitory/residence	0.27	1.06	0.02	0.26	0.797	0.61	1.63
	Worked a part-time job	-1.44	1.06	-0.08	-1.35	0.179	0.99	1.02
	Place where the student was born and raised	3.82	1.30	0.22	2.95	0.004	0.51	1.94
	The house had enough rooms per number of siblings	1.96	0.91	0.13	2.15	0.033	0.85	1.17
	Number of hours of social media connection	-0.27	1.59	-0.01	-0.17	0.867	0.94	1.06
	Study routine	-3.18	0.89	-0.20	-3.59	< 0.001	0.93	1.07
	Parents' level of education	0.29	0.52	0.03	0.55	0.582	0.93	1.07
	Household income	0.21	1.05	0.01	0.20	0.845	0.79	1.26
**State**^**b**^	Gender	0.18	0.78	0.01	0.23	0.818	0.90	1.11
	Academic year	-3.56	0.81	-0.26	-4.42	< 0.001	0.86	1.16
	Need to commute to the university	0.64	1.25	0.05	0.51	0.607	0.34	2.94
	Living in a dormitory/residence	0.08	1.33	0.01	0.06	0.951	0.30	3.30
	Place where the student was born and raised	0.86	1.17	0.06	0.73	0.463	0.50	1.99
	Living area	-1.87	0.91	-0.12	-2.04	0.042	0.85	1.18
	Study routine	-1.67	0.77	-0.12	-2.15	0.032	0.94	1.07
	The house had enough rooms per number of siblings	1.41	0.79	0.10	1.79	0.075	0.88	1.13
	Household income	0.36	0.92	0.02	0.39	0.699	0.80	1.25
**Combined**^**c**^	Academic year	-4.62	1.52	-0.18	-3.04	0.003	0.88	1.14
	Place where the student was born and raised	4.53	2.24	0.16	2.02	0.044	0.50	2.01
	Presence of a chronic disease	6.73	3.18	0.12	2.12	0.035	0.98	1.02
	Experience addiction issue	-8.55	3.95	-0.12	-2.17	0.031	0.93	1.07
	Living area	-1.52	1.74	-0.05	-0.87	0.383	0.85	1.17
	The house had enough rooms per number of siblings	3.69	1.51	0.14	2.44	0.015	0.88	1.14
	Need to commute to the university	0.36	2.40	0.01	0.15	0.881	0.33	2.99
	Living in a dormitory/residence	-0.14	2.53	-0.01	-0.06	0.955	0.30	3.30
	Study routine	-4.88	1.48	-0.18	-3.31	0.001	0.93	1.07
	Household income	0.40	1.76	0.01	0.23	0.820	0.80	1.25

*B* unstandardized coefficients, *SE* standard error, *β* standardized coefficients, *t* t statistics, *p p*-value, *VIF* variance inflation factor

^a^The adjusted R^2^ was 0.14 and the Durbin-Watson ratio was 1.68

^b^The adjusted R^2^ was 0.13 and the Durbin-Watson ratio was 1.63

^c^The adjusted R^2^ was 0.15 and the Durbin-Watson ratio was 1.60

## Discussion

Over the past few years, there have been many calls to promote resilience among practicing and future nurses as an essential component of nurses’ success and abilities to cope with clinical demands in practice [17, 31, 32]. Patients expect to receive healthcare from competent, kind, and compassionate nurses. However, when practicing and future nurses are burnt-out and exhausted, they are less likely to provide care compassionately. Resilience can be characterized as a “trait” or as a “state”. Additionally, resilience can be characterized as a combination of both [13]. In this study, trait, state, and combined resilience scores were assessed among nursing students in Palestine for the first time. In this study, nursing students showed relatively high trait, state, and combined resilience as indicated by their scores obtained on the pre-validated scales. Additionally, predictors of high and low resilience scores were also identified using multiple linear regression models. The findings of this study could be informative to decision and policymakers in academia, student, and nursing professional groups who could be interested in promoting resilience among nursing students.

In this study, the majority of the nursing students reported high prosocial, cognitive, interpersonal, and school functioning (factor 1, items: 1, 3, 8, 9, 16–18) on the Trait Resilience Scale. Nursing students expected themselves to help others, be alert and physically active, have warm positive relationships with others, and persist in actions until success. The findings of this study might substantiate those reported previously that trait resilience can be developed and promoted among nursing students [5]. Traits of resilient individuals include compassionately dealing with others, their sense of having control over their environment, proactively tackling challenges, and solution-seeking behavior in times of adversity. These traits are essential for nursing students to succeed in their academic, training, and future professional careers [5, 33]. Probably, nurse educators need to help nursing students interpret their experiences positively and constructively. This can help students develop resilience, learn from experience, grow stronger, and care for patients compassionately.

The findings of this study showed that the nursing students reported moderate to high personal relationships, role models, and social support (factor 2, items: 5–7 and 13–16). Family support and having someone to turn to in times of adversity were shown to be important in developing resilience [22, 23]. Having a pleasant personality and maintaining warm positive relationships with others could help resilient students to bounce back and carry on with life/professional activities after a time of adversity [5, 13, 19, 22, 23]. During their on-site training, nursing students experience difficulties and witness the sufferings and death of patients. Therefore, nurse educators should promote resilience among students to overcome adversities, consider these adversities as opportunities to learn from, grow stronger, and move on with their life/professional activities [5, 17, 34].

The nursing students in this study reported moderate to high healthy self-perception in self-regulation (factor 3, items: 2, 4, 10–12). Resilient students believe in themselves and often report high self-esteem, remain calm in times of adversity, learn how to handle stressful experiences, and remain an optimist in learning from difficult experiences. These traits are featured in the desired traits of resilient nurses [35, 36]. The findings of this study might add to the literature reporting on resilience among future nurses.

The majority of the nursing students in this study reported high state resilience related to factor 1 (items: 3, 5–6, 9–13). Self-regulation and self-capacity are vital components of resilience. Similarly, the majority of the nursing students reported high state resilience related to factor 2 (items: 1–2, 4, 7–8, 14–15). Previous studies have shown that resilience among nursing students was influenced by interpersonal skills, self-regulation skills, and positive tendencies [37, 38]. It has been argued that these skills contribute to success in academic and professional life [39]. Previous studies have shown that self-regulation can be improved by learning relaxation techniques like meditation, mindfulness, and guided imagery [40]. Learning to control emotions in stressful times can help nursing students develop resilience and learn from difficult experiences. During their on-site training, nursing students might encounter many stressful experiences. Therefore, learning to control their emotions, learning from experiences, and providing care with compassion would be important for their practice.

After controlling for confounding factors, having a study routine on daily basis predicted higher trait, state, and combined resilience scores. These findings were not surprising as a recent meta-synthesis of qualitative studies reported that resilient nurses recognized and acknowledged signs of adversities and strived to develop themselves, foster positive attitudes towards the different aspects of life, developed personal strategies to overcome adversities, planned their lives for a better future, and higher built self-esteem [14]. In this study, higher trait resilience scores were predicted by favorable living conditions including living in a house with enough rooms per sibling, living in Israeli-seized areas, and enjoying better health. Additionally, higher trait resilience scores were also predicted by experiencing addiction issues. Those nurses had personal traits that help them cope with adversities and achieve good adjustments. The findings of this study were consistent with those reported in previous studies [5, 10, 11, 14, 17, 21]. Probably, those students were influenced by favorable family routines and the availability of more support [5, 38]. A previous study showed that nursing students who belonged to high socioeconomic classes reported higher resilience and psychological well-being compared to nursing students of low socioeconomic classes [41]. On the other hand, the findings of this study showed that higher state resilience was predicted by being in the first academic year and living in urban areas. State resilience refers to the cognitive, affective, and motivational potentials of nursing students. State resilience was thought to be relatively malleable and adaptive in different social-ecological contexts. Apparently, the nursing students who lived in urban areas had to adapt to different societal complexities compared to the students who lived in rural areas. On the other hand, the findings of this study contradicted with a previous study in Australia in which resilience levels did not differ between junior and senior nursing students [42].

### Strengths and limitations of the study

The present study has several strengths and limitations that need to be considered when interpreting the findings. First, this study was the first to assess resilience among nursing students in Palestine. The findings of this study could add to the existing literature on resilience among nursing students worldwide, notably, in developing countries. Second, the study was conducted at the main university in Palestine. The university has a large number of nursing students that contribute to a significant proportion of the future workforce of nurses practicing in Palestine. Third, the sample recruited in this study was diversified in terms of representation of both genders, students in different academic years, from different regions, and socioeconomic classes. This diversity might have improved the external validity of the findings and might allow extrapolation of the findings to the entire population of nursing students in Palestine. Fourth, resilience was assessed as a trait, state, and combined in this study. The tools used to assess resilience were previously validated. Additionally, the tools were revalidated in a pilot test that was conducted before the larger study to ensure the tools were valid and reliable in the study settings.

On the other hand, this study has some limitations. First, the sample size was relatively small. The sample size was calculated using a 90% CI. This limitation could have been avoided if the sample size was calculated using a 95% CI. Second, the nursing students were recruited from one university. Recruiting nursing students from other universities should have strengthened and improved the external validity of the findings. Third, social desirability bias cannot be ruled out in this study. As the nursing students had to rate some of their traits, some students could have overrated their traits. Fourth, we cannot rule out recall bias in this study. As the nursing students had to recall memories of the past, we cannot rule out recall bias in this study. Fifth, 42.8% of the students who responded to the questionnaire were in the first academic year. This could have led to participation bias. Sixth, analysis of the combined resilience scores could be associated with an increased alpha error. Seventh, although resilience is thought to be shaped by the sociodemographic, economic, and academic variables of the nursing students, a causal relationship cannot be proven in this cross-sectional study. Another prospective study should be conducted to see how resilience changes as students advance in their academic years. Nurse educators and other stakeholders are recommended to take into planning how to support nursing students to improve their resilience and transition into their future nursing practice.

## Conclusion

In conclusion, undergraduate nursing students in Palestine reported relatively high trait, state, and combined resilience scores. There was a significant moderate positive correlation between state resilience scores and trait resilience scores. Higher trait, state, and combined resilience scores were predicted by having a study routine on daily basis. Trait and combined resilience scores were predicted by the absence of chronic diseases, experiencing addiction issues, living in Israeli seized areas, and living in a house with enough rooms per sibling. State and combined resilience scores were predicted by being in the first academic year and state resilience scores were predicted by living in urban areas. More studies are still needed to investigate the relationship between resilience scores, perceived well-being, willingness to care, and the future success of nursing students in Palestine.

## Data Availability

All data relevant to this work are included within the manuscript or in the additional file as supplementary materials.

## Abbreviations

CI: Confidence interval
GPA: Grade point average
IP: Internet Protocol
IRB: Institutional Review Board
SD: Standard deviation
STROBE: Strengthening the Reporting of Observational Studies in Epidemiology
